# Vertical Distribution of Arthropod Interactions Within Turfgrass

**DOI:** 10.1093/jisesa/ieac050

**Published:** 2022-09-09

**Authors:** Fawad Z A Khan, Shimat V Joseph

**Affiliations:** Department of Entomology, University of Georgia, 1109 Experiment Street, Griffin, GA 30223, USA; Department of Entomology, University of Georgia, 1109 Experiment Street, Griffin, GA 30223, USA

**Keywords:** clay model, canopy, conservation biological control, predation, impression

## Abstract

Arthropod predators are abundant in turfgrass systems, and they play an important role in managing pests. Understanding the vertical distribution of predation is critical to developing cultural strategies that enhance and conserve predatory services. However, little is known on how the predation is vertically distributed within the turfgrass canopy. Thus, the objective of this study was to determine the vertical distribution of predation within the turfgrass canopy. Clay models were used to emulate the general appearance of Noctuidae caterpillars, to estimate the predatory activity. The choice and no-choice experiments were conducted by placing clay models at 2.54, 5.08, and 7.62 cm from the thatch surface and denoted as lower, intermediate, and upper levels, respectively, within turfgrass canopy. The predator-mediated impressions, paired mark, scratch, deep cut mark, deep distortion, prick, dent, stacked surface impression, scooped mark, granulation, and U-shaped mark, were identified on clay models. The incidence and severity of impressions were significantly greater on clay models placed at the lower canopy level than on those placed at the intermediate and upper canopy levels in the choice and no-choice experiments (*P* < 0.05). Thus, predators are more likely to find their prey at the soil level. This information can be used to refine management strategies, such as mowing height and insecticide use for effectively managing soil-borne and foliar-feeding arthropod pests and beneficial arthropods.

Turfgrass is an important component of residential lawns, golf courses, athletic fields, and public parks and turfgrass pests reduce the aesthetic and commercial value of turfgrass (Potter and Braman 1991). Based on the activity of various stages of arthropod pests, turfgrass is categorized into three zones, 1) stem and leaves, 2) thatch, and 3) soil and root region (Williamson et al. 2015). Most of the pests fall into these zones. Moreover, how arthropod communities are structured around the arthropod pest populations is unclear. The arthropod communities might be same across turfgrass systems but their interactive activities could be more intense in one or more zones within turfgrass canopy. For example, the fall armyworm, *Spodoptera frugiperda* (JE Smith) and, black cutworm, *Agrotis ipsilon* (Hufnagel) (both Noctuidae: Lepidoptera) are the major turfgrass pests, and larval stages of these pests are most active in the stem and foliar or thatch zones of turfgrass (Watschke et al. 2013). The behavior of arthropod predators, particularly foraging and feeding behavior within the zones of turfgrass, determines the fate of natural pest management. For example, many formicids are active within the turfgrass canopy, and they effectively encounter and prey on larval stages of *A. ipsilon* (López and Potter 2000) and *S. frugiperda* (Braman et al. 2002, Khan and Joseph 2022). However, the vertical distribution of predatory behavior and activity within the turfgrass canopy is poorly understood. Thus, the hypothesis of the current study was that the interactive activity of arthropod communities may be confined to one or more zones within the turfgrass canopy. This information can enhance our understanding how to improve or refine the integrated pest management programs against the major arthropod pests in turfgrass as turfgrass is high value commodity in the United States (Haydu et al. 2008, Wolfe and Stubbs 2020).

Clay models simulating prey species have been used to estimate and document predation rates in urban, forest, and cropping ecosystems (Molleman et al. 2016, Seifert et al. 2016, Ferrante et al. 2017, Roels et al. 2018, Zvereva et al. 2019, Nason et al. 2021). In these studies, the prey models were deployed in the specific environment and recovered after specified intervals, and the impressions created on the models were evaluated (Stuart et al. 2012, Rößler et al. 2018, Witwicka et al. 2019, Khan and Joseph 2021a). The types of impressions on the clay model surface provide information about the types of organisms, such as arthropods, mammals, birds, and reptiles, that interacted with the models (Low et al. 2014, Khan and Joseph 2021a). The clay model tool has not been utilized fully to determine predation rates in turfgrass.

Turfgrass management involves maintaining turfgrass at a specific mowing height, which varies with factors, such as the aesthetic needs, growing climatic zone, the utility of turfgrass, and the grass genotype installed. In Georgia (USA), e.g., the recommended mowing height for the bermudagrass [*Cynodon dactylon* (L.) Pers.] is 2.5– 5cm, while it is 5–7.5 cm for the St. Augustinegrass [*Stenotaphrum secundatum* (Walt.) Kuntze] (Waltz et al. 2020). Taller turfgrasses provide refugia sites for predatory arthropods, improving conservation biological control (Dobbs and Potter 2014). For example, greater predation of *Ataenius spretulus* (Haldeman) (Coleoptera: Scarabaeidae) grubs has been reported on rough of annual bluegrass (*Poa annua* L.) maintained at 5 cm height than on fairways maintained at 1.5 cm height in a golf course (Jo and Smitley 2003). Similarly, the abundance of rove beetles (Coleoptera: Staphylinidae) and spiders (Araneae) increased with an increase in the mowing height of cool-season turfgrass (Dobbs and Potter 2013). However, the vertical distribution of predator activity patterns within the canopy of warm-season turfgrass is still unclear. Thus, the objective of this study was to determine the potential predation pressure at different heights of the turfgrass after placing the clay models at lower, intermediate, and upper canopy levels of turfgrass.

## Materials and Methods

### Study Site and General Methods

In 2020, experiments were conducted on the ‘Tifway’ bermudagrass field at the University of Georgia, Griffin, Georgia, USA. To determine the vertical distribution of the predation within the turfgrass canopy, the turfgrass was maintained at 8 cm high based on the University of Georgia recommendations and was mowed at 7 d intervals for 8 wk. The turfgrass was irrigated 30 min daily from 5:30 to 6:00 a.m. for 8 wk. The fertilizers and pesticides were not applied. The turfgrass site was infested with weeds, such as southern crabgrass, *Digitaria ciliaris* (Retz.), white clover, *Trifolium repens* L., which covered <10% of the experimental area. The 2,604.73 m² experimental site was part of the 80,042.21 m² open turfgrass field with no trees within a 70 m radius.

### Clay Model

The clay models were prepared using green, nontoxic clay (Sculpey III, Polyform Products, Elk Grove Village, IL). The green-colored clay was selected to mimic an undefended prey. The stability of this clay product was proven under summer temperatures (Roels et al. 2018). The green-colored clay models effectively captured predatory interactions, and as a result, the impressions were visible on the clay surface (Low et al. 2014, Sam et al. 2015, Roels et al. 2018, Long and Frank 2020, Khan and Joseph 2021a). A 30 × 4 mm (length × diameter) model, simulating the late instar caterpillar pest, such as fifth instar of *S. frugiperda* larva, was prepared by rolling the clay on a smooth wooden surface using a piece of a 10 cm × 5 cm clear acrylic sheet (Khan and Joseph 2021a). The consistency of the cylindrical shape of the model was ensured by regularly measuring the diameter of the rolled clay using a Vernier caliper (model #1468417; General UltraTech, Friendswood, TX). The rolled clay was then cut into 4 × 30 mm (diameter × length) cylinders. The clay models were molded to a C-shape to resemble the stationary posture of a fifth-instar *S. frugiperda*. This C-shaped clay model was then glued on a 7.62 × 1.79 cm (length × width) weatherproof paper card (JL Darling, Tacoma, WA) using ~300 mg of silicone glue (Arrow Fastener Co., LLC., Saddle Brook, NJ) by using an electric hot melt glue gun.

### Experimental Design

**Fig. 1.**
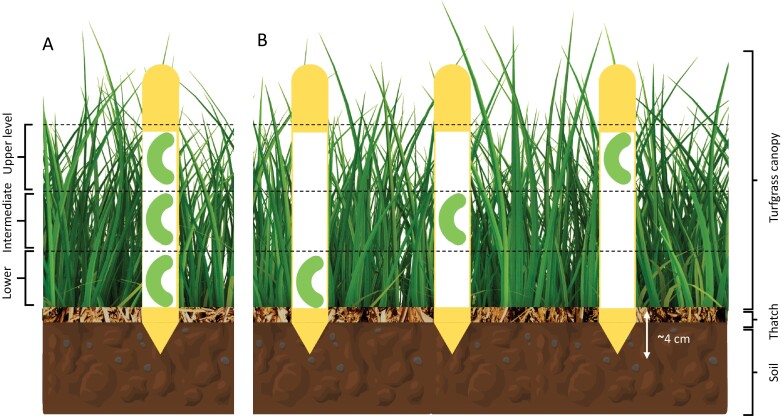
Schematic diagram of (A) choice, (B) no-choice study. The figure shows the placement of various treatments within the turfgrass canopy.

As described in the Clay Model Section, the clay models were prepared and glued to paper cards, and the cards with the clay models were then glued to a 15.24 × 1.79 cm × 0.17 cm (length × width × height) wooden stake. Both choice and nonchoice studies were conducted. For the choice study, three clay models were glued individually to a wooden stake at three heights of 2.54, 5.08, and 7.62 cm from the thatch surface, and the three height or level treatments were denoted as lower, intermediate, and upper canopy levels, respectively (Fig. 1A). The height was determined by measuring the length from the thatch surface to the center of each clay model attached to the stake. The tip of the lower clay model treatment was at ~0 cm from the thatch surface upon deployment. For the no-choice study, only one clay model was attached to a wooden stake at one of the three heights (2.54, 5.08, or 7.62 cm) (Fig. 1B). The clay model was placed at a specific height on the wooden stake, and it served as the experimental unit. The treatments of the choice experiment were arranged in a randomized complete block design with 30 replications. For the no-choice experiment, the treatments were replicated 10 times. The treatments in the choice and no-choice experiments were deployed 6 m away from the edge of the field and were 3 m apart within and between the blocks. The clay models were exposed to turfgrass arthropods for 24 h. The choice and no-choice experiments were simultaneously conducted from 8 to 11 July 2020 on the same experimental site and were repeated from 2 to 5 September 2020. The experiments were conducted in July and September because most insects are active in summer, and the *S. frugiperda* problem is common in turfgrass from July to September. July and September were randomly selected for repetition and were not associated with the hypothesis of the current study.

### Clay Model Evaluation

Clay models were recovered from the field, transported to the laboratory, and stored at room temperature (21°C) until evaluation. Khan and Joseph (2021b) characterized nine impression types on the clay models. The impressions found on the clay models were evaluated using the impressions characterized by Khan and Joseph (2021b) using a dissecting stereomicroscope (40×) (M3, Wild Heerbrugg AG, St. Gallen, Switzerland). The impression types: paired mark, scratch, deep cut mark, prick, dent, and U-shaped mark, were quantified. The U-shaped mark was only observed on September choice experiment. The impression types: deep distortion, stacked surface impression, scooped mark, and granulation, were evaluated as a percentage of damaged clay model surface area. To understand the severity of damage, percentages of model surface area affected were estimated using a scale system (0 = 0% impressions, 1 = 1–10%, 2 = 11–20%, 3 = 21–30%, 4 = 31–40%, 5 = 41–50%, 6 = 51–60%, 7 = 61–70%, 8 = 71–80%, 9 = 81–90%, and 10 = 91–100% of surface exhibiting at least one impression type). We evaluated and documented various impression types within the turfgrass canopy because differences in types of impressions across vertical zones could reflect types of predators and their general behavior when interacting with prey.

### Statistical Analyses

All the data analyses were performed in SAS (SAS Institute 2012). For the prey placement choice and no-choice experiments, the numbers of impressions by impression type on the clay model treatments were subjected to analysis of variance (ANOVA) using the PROC GLIMMIX procedure in SAS. The procedure used a generalized linear mixed model with negative binomial distribution and log link function. The treatments were the placement heights of the models in the turfgrass canopy and served as the fixed effect, and replications served as the random effect. The estimation method was maximum likelihood with the Laplace approximation. Because data were analyzed using a generalized linear model with a log-link function, the data were not assessed for normality. The means were separated using Tukey–Kramer multiple comparison test (*P* < 0.05). The interaction intensity data recorded as scale values and those impression data recorded as percentages were arcsine square-root transformed before running the PROC GLM procedure. The means were separated using Tukey’s studentized range HSD test. The means and standard errors of the variables were calculated using the PROC MEANS procedure in SAS.

## Results

Of 720 clay models exposed at three levels, 661 had predatory impressions, which suggested 91.8% overall predation in choice and no-choice experiments (conducted in July and September 2020). The predator impression types have been characterized (Khan and Joseph 2021a,b,^2022^). Predator densities can vary through the growing season (Joseph and Braman 2009, Singh 2020). All the clay models were recovered after exposure.

### Choice Experiment

**Fig. 2.**
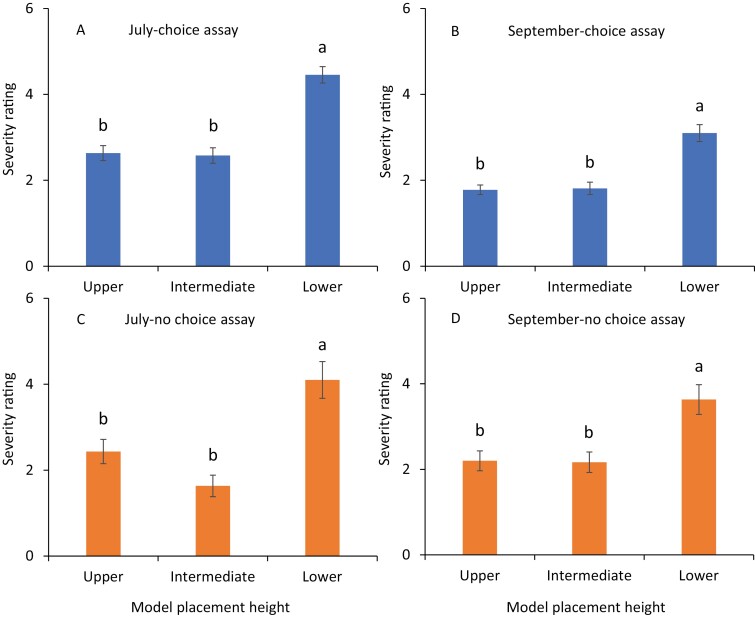
Mean (±SE) severity rating observed on clay models in choice experiments during (A) July, (B) September 2020 as well as no-choice experiments during (C) July and (D) September 2020. The same letters above the bars denote no significant difference (Tukey’s Studentized Range HSD test; *P* < 0.05).

**Fig. 3.**
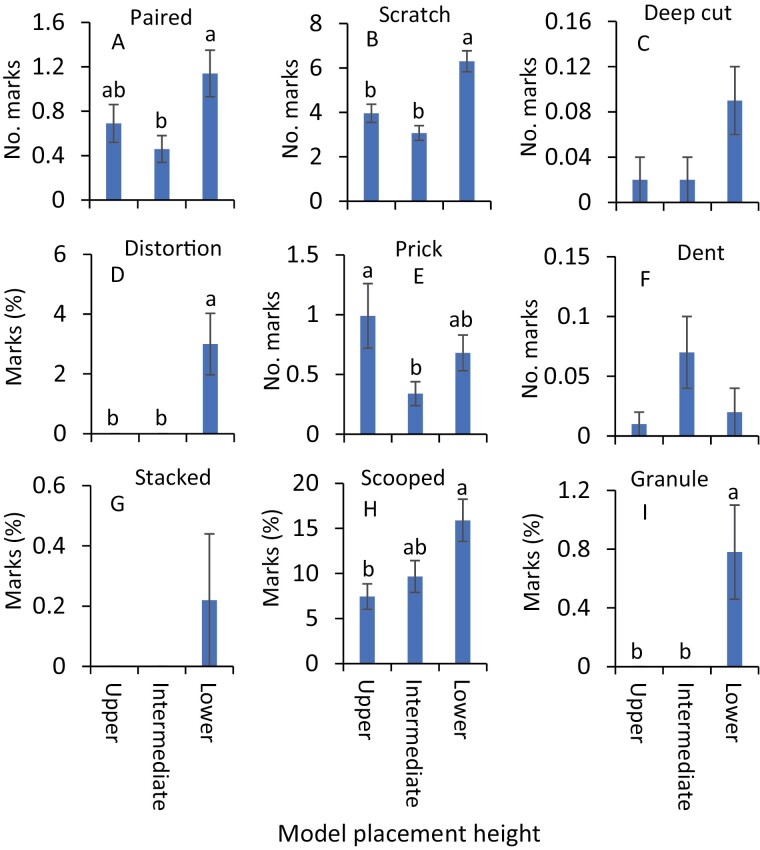
Mean (±SE) impressions (A) paired marks, (B) scratches, (C) deep cut marks, (D) deep distortions, (E) pricks, (F) dents, (G) stacked surface marks, (H) scooped marks, and (I) granulations observed on clay models in choice experiments during July 2020. The same letters above the bars denote no significant difference (Tukey’s Studentized Range HSD test; *P* < 0.05). Where no significant differences were observed, no letters are included.

In July, impressions were significantly more severe on the clay models at the lower canopy level than at intermediate and upper canopy levels (*F* = 35.4, df = 2, 238, *P* < 0.001, Fig. 2A). Ten distinct impression types, paired mark, scratch, deep cut mark, deep distortion, prick, dent, stacked surface impression, scooped mark, and granulation were observed on the clay models. In July, the numbers of paired marks were significantly greater on the clay models at the lower canopy level than those at the intermediate canopy level (Fig. 3A; Table 1). Similarly, the numbers of scratches were significantly greater on the clay models placed at the lower canopy level than those placed at intermediate and upper canopy levels (Fig. 3B; Table 1). Significantly greater percentages of deep distortions and granulations were observed at the lower canopy level than those at intermediate and upper canopy levels (Fig. 3D and I; Table 1). The scooped marks were significantly greater on clay models placed at the lower canopy level than those on the other two upper levels (Fig. 3H; Table 1). In contrast, pricks were significantly greater on the models at the upper canopy level than on the lower and intermediate canopy levels (Fig. 3E; Table 1). The numbers of deep cuts and dents and the percentages of stacked surface marks were not significantly different on clay models placed among various heights (Fig. 3C, F, and G; Table 1).

**Table 1.** Analysis of variance of impressions (number or percentage) recorded on clay models deployed at different heights in choice and no-choice experiments

	Model placement	Impressions								
		Paired marks^a^	Scratches^a^	Deep cut marks	Deep distortions^b^	Pricks^a^	Dents^a^	Stacked surface marks^b^	Scooped marks^b^	Granulations^b^
*Choice*										
July 2020										
*F*; df		5.6; 2, 238	14.8; 2, 238	2.4, 2, 238	9.1; 2, 238	3.6; 2, 238	1.9; 2, 238	1.0; 2, 238	5.4; 2, 238	6.1; 2, 238
*P*		0.004	<0.001	0.095	<0.001	0.029	0.148	0.369	0.005	0.003
September 2020										
*F*; df		11.0; 2, 238	6.9; 2, 238	0.5; 2, 238	0.5; 2, 238	2.9; 2, 238	1.0; 2, 238	0.9; 2, 238	1.9; 2, 238	1.0; 2, 238
*P*		<0.001	0.001	0.625	0.601	0.057	0.372	0.396	0.155	0.369
*No-choice*										
July 2020		11.0; 2, 78	5.0; 2, 78	2.6; 2, 78	3.8; 2, 78	5.8; 2, 78	0.1; 2, 78	1.0; 2, 78	0.9; 2, 78	2.4; 2, 78
*F*; df		<0.001	0.009	0.083	0.028	0.004	0.897	0.3727	0.426	0.094
*P*										
September 2020		6.0; 2, 78	1.1; 2, 78	0.3; 2, 78	2.2; 2, 78	0.6; 2, 78	0.8; 2, 78	–	–	–
*F*; df		0.004	0.322	0.725	0.113	0.555	0.435	–	–	–
*P*										

Means within a column followed by the same letters are not significantly different (Tukey–Kramer multiple comparisons test at *P* < 0.05).

Means within a column followed by the same letters are not significantly different (Tukey’s Studentized Range HSD test at *P* < 0.05).

**Fig. 4.**
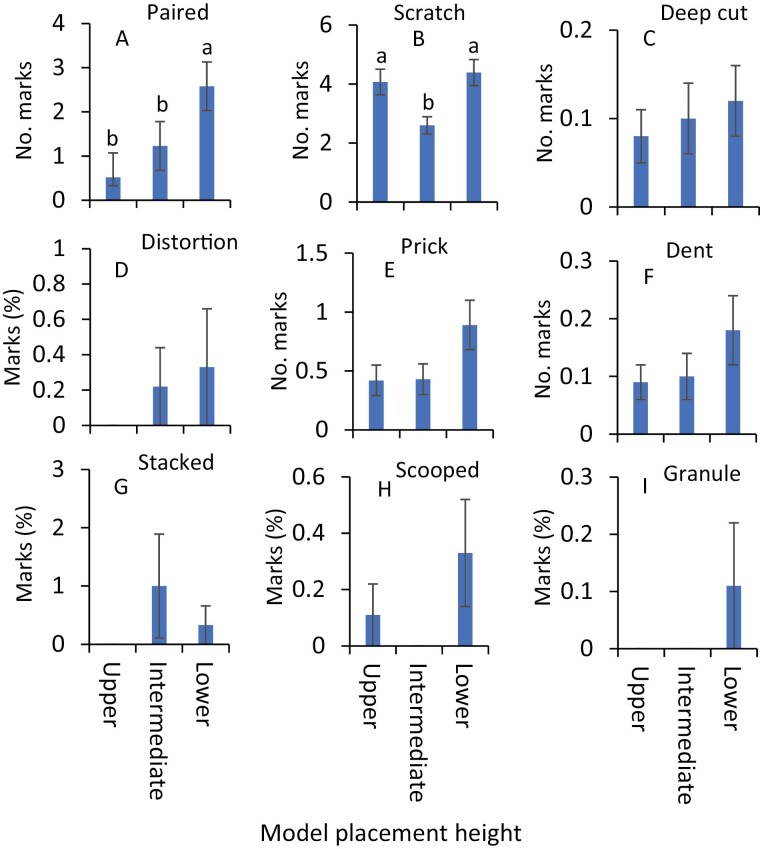
Mean (±SE) impressions (A) paired marks, (B) scratches, (C) deep cut marks, (D) deep distortions, (E) pricks, (F) dents, (G) stacked surface marks, (H) scooped marks, and (I) granulations observed on clay models in choice experiments during September 2020. The same letters above the bars denote no significant difference (Tukey’s Studentized Range HSD test; *P* < 0.05). Where no significant differences were observed, no letters are included.

In September, the scale values of impressions were more severe on the clay models at the lower canopy level than those at intermediate and upper canopy levels (*F* = 20.3, df = 2, 238, *P* < 0.001, Fig. 2B). The numbers of paired marks were significantly greater on clay models at the lower canopy level than at intermediate and upper canopy levels (Fig. 4A; Table 1). The numbers of scratch marks on the clay models at the lower and upper canopy levels were significantly greater than those on the intermediate canopy level (Fig. 4B; Table 1). The numbers of deep cut marks, pricks, and dents, as well as the percentages of deep distortion, stacked surface marks, scooped marks, and granulations on the clay models, were not significantly different among placement heights (Fig. 4C–I; Table 1).

### No-choice Experiment

**Fig. 5.**
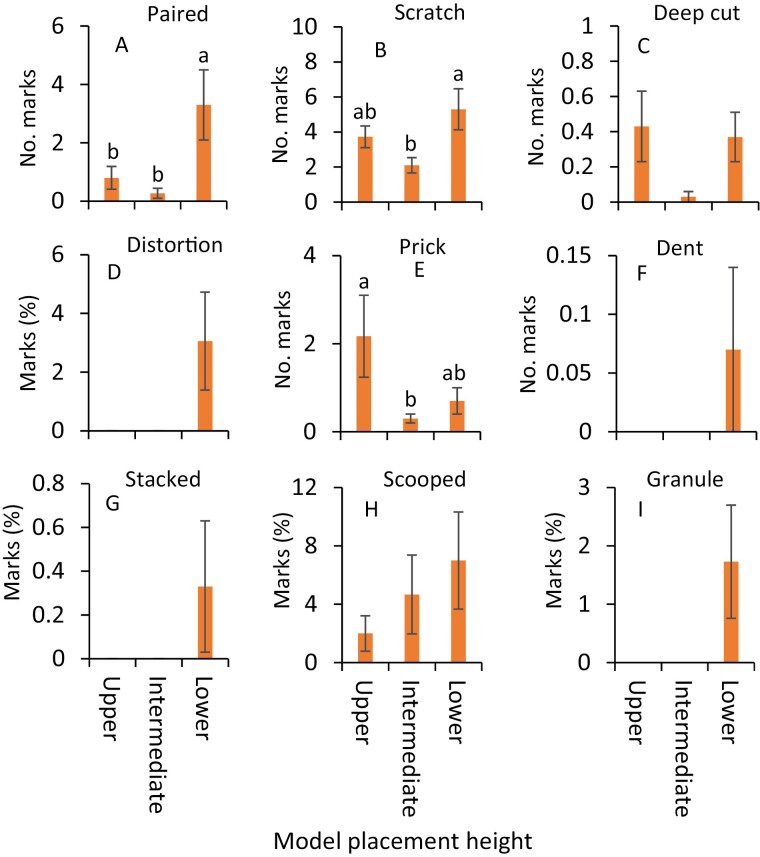
Mean (±SE) impressions (A) paired marks, (B) scratches, (C) deep cut marks, (D) deep distortions, (E) pricks, (F) dents, (G) stacked surface marks, (H) scooped marks, and (I) granulations observed on clay models in no-choice experiments during July 2020. The same letters above the bars denote no significant difference (Tukey’s Studentized Range HSD test; *P* < 0.05). Where no significant differences were observed, no letters are included.

Nine distinct impression types were identified in the no-choice experiments, and they were paired mark, scratch, deep cut mark, deep distortion, prick, dent, stacked surface impression, scooped mark, and granulation. In July, the scale values related to the severity of impressions on the clay models were significantly greater at the lower canopy level than those placed at the intermediate and upper canopy levels (*F* = 14.0, df = 2, 238, *P* < 0.001, Fig. 2C). The numbers of paired marks were significantly greater on clay models at the lower canopy level than those at the intermediate and upper canopy levels (Fig. 5A; Table 1). Significantly greater numbers of scratches were observed on clay models at the lower canopy level than at the intermediate canopy level (Fig. 5B; Table 1). In contrast, the numbers of pricks were significantly greater on the clay models at the upper canopy level than those at the intermediate canopy level (Fig. 5E; Table 1). The numbers of other impressions, deep cut marks, and dents, as well as the percentages of deep distortion, stacked surface impressions, scooped marks, and granulation, on the clay models, were similar across the canopy levels (Fig. 5C, D, F, G, H and I; Table 1).

**Fig. 6.**
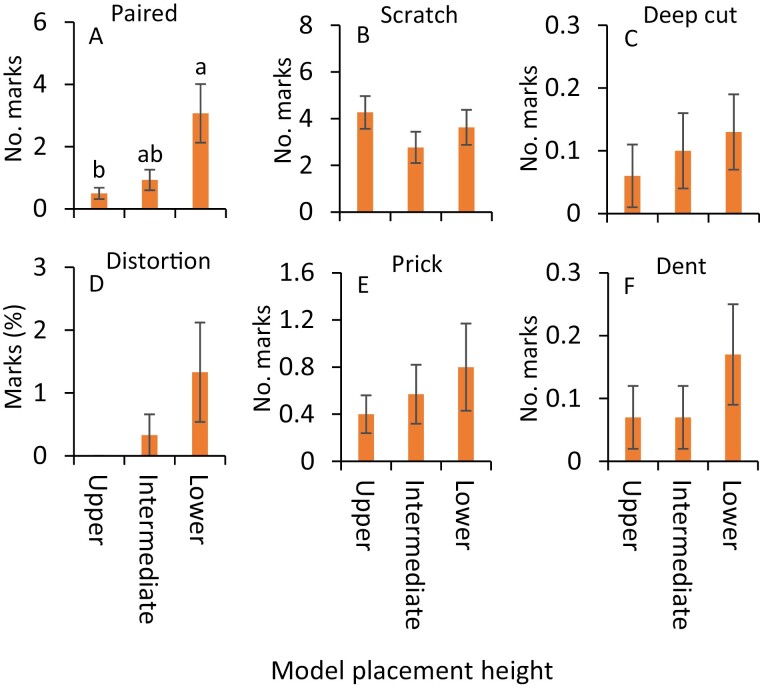
Mean (±SE) impressions (A) paired marks, (B) scratches, (C) deep cut marks, (D) deep distortions, (E) pricks, and (F) dents observed on clay models in no-choice experiments during September 2020. The same letters above the bars denote no significant difference (Tukey’s Studentized Range HSD test; *P* < 0.05). Where no significant differences were observed, no letters are included.

In September, the severity of impressions was significantly greater on the clay models at the lower canopy level than those placed at the intermediate and upper canopy levels (*F* = 8.7, df = 2, 238, *P* < 0.001, Fig. 2D). The clay models placed at the lower canopy level captured significantly greater numbers of paired marks than those placed at the upper canopy level (Fig. 6A; Table 1). The numbers of other impression types, including scratches, deep cut marks, pricks, and the percentages of deep distortion, were not significantly different among canopy levels (Fig. 6B–F; Table 1).

## Discussion

The results showed that the clay models placed near the thatch surface captured most impressions, which suggests arthropod predators are more active on the soil or thatch surface in turfgrass. Both predators and these lepidopteran larvae share nocturnal feeding habits in turfgrass (Sparks 1979, Khan and Joseph 2021b). This suggests that predators in the turfgrass are likely to encounter noctuid caterpillars, such as larval stages of *A. ipsilon* and *S. frugiperda*, and enhance biological control. In addition to these consumptive effects, active foraging activity of predators on the thatch surface can induce nonconsumptive effects, such as disrupting mating, oviposition, or dispersal behavior of insect pests, in addition to direct predation (Dupuy and Ramirez 2019).

The predatory interactions were more severe at the thatch surface than at the upper strata of the turfgrass canopy. This finding suggests that either the densities of a predator species were greater on the thatch surface or a diverse group of predator species was active on the thatch surface of the turfgrass canopy. In addition, some of the impression types were more severe than others on the thatch surface. The paired marks were the most abundant impression type on the thatch surface than other types of impression. Previously, the paired marks were documented on the clay models when they were exposed to carabids, formicids, anisolabidids, labidurids, and lycosids (Khan and Joseph 2021a). Also, Braman et al. (2002) showed that formicids were the most abundant predators in turfgrass and were observed to consume the egg and larval stages of *S. frugiperda*. It is possible that some of the impressions are caused by avian predators and wasps as indicated in Low et al. (2014)

Although arthropod interactions were most abundant at the thatch surface of the turfgrass, the methodology of the current study brings a few limitations. First, all the clay models have been vertically positioned, assuming the larval climbing posture at the various levels of the turfgrass canopy. It is unclear if the vertical placement method of the clay model reduced or overestimated the interaction events from the approaching predators. Previous turfgrass field studies estimated predation by placing the clay models horizontally on the thatch or the top of the grass canopy (Khan and Joseph 2021b). However, it is unclear which placement positions (vertical or horizontal) would yield more predation rates in turfgrass. Second, because the clay models were attached to one side of the wooden stake, it is unclear if it reduced the attack rates as exposure of clay models to predators was restricted from one side. Third, the experiments were conducted on bermudagrass, and it is unclear if predator behavior varies by turfgrass genotype and their growth pattern, leaf texture, and management practices, as Joseph and Braman (2009) showed that the occurrence and abundance of the beneficial arthropods could vary by turfgrass types. Finally, the experiment was performed on the turfgrass maintained at a constant height. A taller or shorter grass height may influence the abundance of arthropod predators in the turfgrass (Dobbs and Potter 2013).

The data show that the predation rates were higher at the thatch surface within the turfgrass canopy than in the upper regions of the turfgrass. The results also indicate that the placement of clay models at the thatch surface within the turfgrass canopy leads to more interactions. Dobbs and Potter (2014) showed that staphylinids and Araneae were less abundant in the shorter than in the taller turfgrass. In contrast, the abundance of other predatory groups, such as formicids, remained unaffected by turfgrass height. Thus, more research is warranted to determine how the turfgrass height and other disruptive cultural management practices influence the predation rates in various turfgrass genotypes.

## Author contibutions

FZAK and SVJ were involved in the design of the experiments. FZAK conducted the experiments, analyzed the data, and prepared the original draft of the manuscript.SVJ edited the manuscript and prepared the final version.
